# The plaque reducing efficacy of oil pulling with sesame oil: a randomized-controlled clinical study

**DOI:** 10.1007/s00784-024-06134-y

**Published:** 2025-01-09

**Authors:** Christine Zürcher, Kristian Vukoje, Eva Maria Kleiner, Sophie Martina Kuster, Lisa Katharina Jäger-Larcher, Ingrid Heller, Sigrun Eick, Markus Nagl, Ines Kapferer-Seebacher

**Affiliations:** 1 University Hospital for Conservative Dentistry and Periodontology, Medical University of Innsbruck, Innsbruck, 6020 Austria; 2 Institute of Hygiene and Medial Microbiology, Medical University of Innsbruck, Innsbruck, Austria; 3 University Hospital for Dental Prosthetics, Medical University of Innsbruck, Innsbruck, Austria; 4 Department of Periodontology, School of Dental Medicine, University of Bern, Bern, Switzerland; 5 Institute of Hygiene & Medical Microbiology, Medical University of Innsbruck, Innsbruck, Austria; 6 MB-LAB - Clinical Microbiology Laboratory, Innsbruck, 6020 Austria

**Keywords:** Oil pulling, Sesame oil, Dental biofilm(s), Oral hygiene, Plaque index

## Abstract

**Objectives:**

To compare the plaque reducing efficacy of oil pulling with sesame oil compared to distilled water in a randomized, controlled, examiner-blinded parallel group study.

**Materials and methods:**

Forty probands without advanced periodontal disease of the University Hospital for Restorative Dentistry and Periodontology, Medical University of Innsbruck (Austria) were randomized allocated to test- (sesame oil) or control group (distilled water) and asked to pull daily in the morning for eight weeks with their allotted fluid for 15 min. Rustogi Modified Navy Plaque Index (RMNPI) and gingival bleeding index were assessed at baseline and after four and eight weeks. Plaque samples underwent microbiological analysis.

**Results:**

Pulling with sesame oil was significantly more effective in reducing full mouth RMNPI compared to distilled water after eight weeks (median reduction 18.98% versus 10.49%; *p* = 0.023), and was most pronounced in anterior, buccal, and lingual subscales. On approximal surfaces, significantly higher plaque reduction was found in the test group after four (24.07% versus 14.29%) and eight weeks (16.00% versus 5.36%) of intervention (*p* < 0.05). No significant changes in gingival index and mirobiological analysis could be detected.

**Conclusion:**

Plaque reduction was statistically significantly higher with oil pulling than with distilled water, however, a study bias cannot be ruled out. Further high-quality trials are needed to understand the mechanisms and effectiveness of oil pulling, to finally clarify the evidence.

**Clinical relevance:**

Oil pulling may be recommended as an adjuvant to mechanical dental cleaning. Individuals with keratosis may experience adverse effects.

**Trial registration:**

ClinicalTrials.gov NCT06327841.

**Supplementary Information:**

The online version contains supplementary material available at 10.1007/s00784-024-06134-y.

## Introduction

Oil pulling has its origin in India and is an ancient Ayurvedic practice. It recently gained popularity due to its natural origin, cost effectiveness, and negligible side effects. The procedure involves swirling a tablespoon of oil around in the mouth and pulling it between the teeth for about 20 min. The oil is then spit out, the mouth thoroughly rinsed with warm saline or tap water, and the teeth are then cleaned in the usual way [1, 2]. It is recommended to practice oil pulling with organic and cold-pressed oils. Traditionally, sesame oil or coconut oil is preferred, but also sunflower, olive, rapeseed or other edible oils can be used. Depending on the oil, different compounds such as polyphenols, tocopherols or vitamins with antimicrobial, antioxidant, and immunomodulatory properties can be found [3, 4]. The lignans in sesame oil (sesamin, sesamolin and sesaminol) have powerful antioxidative capacities and enhance the action of vitamin E, C and provitamin A [5, 6]. Coconut oil contains about 50% lauric acid, which has antimicrobial and anti-inflammatory properties [7, 8]. Olive oil contains phytosterols, squalene, vitamins A, E and K and phenolic compounds, which have antimicrobial, antioxidative and immunomodulatory properties [9–11].

Mechanical tooth cleaning is undoubtedly the primary element of home oral hygiene and is essential in preventing biofilm-induced diseases such as caries, gingivitis, and periodontitis. Unfortunately, plaque levels achieved in the general population are modest despite intensive efforts in prevention and developing oral hygiene measures [12]. Dental biofilms are a natural phenomenon from an evolutionary and biological point of view. Recent hypotheses suggest that periodontal pathogenesis is the result of “personalized pathology”, from interactions between a highly diverse commensal microbiota and the host, driven by interactions of genetic and environmental factors [13]. Thus, treatment of oral biofilm-induced diseases should increasingly focus on changes in the composition of the oral microbiome induced by diet, stress modulation and smoking cessation [14]. In this context, oil pulling is said to have several positive effects, not only on biofilm formation but also on microbial composition and immunologic processes. A systematic review including four randomized-controlled trials (RCTs) showed significant reductions in plaque indices after oil pulling [15]. The major mechanisms are said to be the mechanical cleaning capacity of oil due to its high viscosity, saponification, and emulsification [5]. Furthermore, oil coats the dental surface, acting as a viscous barrier against plaque formation and bacteria aggregation [16–18]. However, there are conflicting reports on the effect of oil pulling on plaque and gingival indices. A recent meta-analysis on 25 clinical trials reported on statistically significant improvements of gingival index scores after 7 to 45 days of oil pulling compared to non-chlorhexidine mouthwash, but it was less effective in reducing plaque index scores compared to chlorhexidine [19].

Regarding biofilm composition, single oil pulling studies with different oils showed a reduction in total bacterial counts, specifically in the total count of aerobic microorganisms, or the decline of single microbiological species like *Streptococcus mutans* [20], *Lactobacillus species* [21], and *Candida albicans* [22] in saliva or plaque samples. A systematic review reports about two RCTs detecting significant reduction in salivary bacterial colony counts after oil pulling [15], and a meta-analysis showed the same significant results regarding colony counts, but no significant difference of salivary S*treptococcus mutans* counts between oil pulling and control group [23].

All systematic reviews conclude that the evidence on oral health effects of oil pulling is limited due to the absence of high-quality studies. Therefore, the aim of the present study was to strengthen the scientific evidence on the effect of oil pulling on oral health, with respect to plaque levels and gingival health. The plaque and gingivitis reducing efficacy of oil pulling with sesame oil versus distilled water was evaluated in a randomized, controlled, examiner-blinded clinical trial.

## Materials and methods

The Ethics committee of the Medical University of Innsbruck, Austria, approved the study (ID EK 1117/2022). The study was conducted in accordance with the 1964 Helsinki Declaration and its later amendments. All subjects signed an informed written consent prior to the study enrollment.

### Study subjects

Forty volunteers were recruited at the Department of Dental and Oral Medicine and Cranio-maxillofacial and Oral Surgery of the Medical University of Innsbruck (Austria) from May 4th to June 23rd, 2022. Inclusion criteria were age ≥ 18 years, contractual capability, the presence of ≥ 10 teeth, and community periodontal index of treatment needs (CPITN) grade 1 or 2 [24]. Exclusion criteria were missing consent, CPITN grade 0, 3 or 4, pregnancy or breastfeeding, systemic diseases or conditions that are associated with an increased risk of infection or necessitate concomitant antibiotic therapy with dental treatment, mental and behavioral disorders that impede (verbal) communication, allergy against sesame (oil), intake of antibiotics 6 months prior to or during study duration, intake of medication potentially influencing gingival inflammation or bleeding (e.g. anticoagulants, cortisone), infectious diseases (e.g. HIV, hepatitis B or C), fixed orthodontic appliances, ongoing oil pulling or mouth rinsing, adult guardianship, and insufficient nasal breathing.

### Clinical intervention

Data collection was carried out at the University Hospital for Conservative Dentistry and Periodontology, Medical University of Innsbruck (Austria) from June 22nd to August 22nd 2022. According to computer-generated randomization (Microsoft^®^ Office Excel), probands were allocated either to the test group, who practiced oil pulling with native sesame oil from controlled organic cultivation (Rapunzel Naturkost GmbH, Legau, Germany) or to the control group, designated to rinse with distilled water (Ampuwa Spüllösung, Fresenius Kabi AG, Bad Homburg, Germany). Both liquids were provided in identical amber glass bottles of 500 ml (Rapunzel Naturkost GmbH, Legau, Germany). At the beginning, each subject was asked to attend three appointments. At day one, the probands were informed about the study procedure, they signed an informed consent, and inclusion and exclusion criteria were proved. All individuals were clearly instructed not to change their oral hygiene behaviors during the study period. As a control, daily oral hygiene measures were documented at baseline and at follow-up.

The clinical investigation was done by one blinded investigator (KV). Gingival bleeding index (GBI) was assessed dichotomously at six sites per tooth 25 s after irritating the gingival margin with a periodontal probe (Parodontometer PCP12, Hu-Friedy Mfg. Co., LLC, Chicago, USA). Third molars, carious teeth and implants were excluded. Gingival index was calculated as percentage of bleeding sites to measured sites. The primary outcome measure Rustogi Modified Navy Plaque Index (RMNPI) [25] was investigated after plaque disclosing with 2Tone (Young, Earth City, Mo, USA). The index divides buccal and lingual surfaces into nine areas (A to I) that are scored for the presence (score = 1) or absence (score = 0) of plaque. Assessing the whole mouth (areas A–I), interdental (areas D and F), and gingival margin (areas A–C) plaque presence, provides insights about plaque geography as each area can be evaluated separately. Third molars and carious teeth were excluded from the evaluation, whereas teeth with fillings, inlays, onlays, or crowns were included. RMPNI is calculated as percentage of biofilm adhering sites to measured sites.

Supragingival plaque samples were taken from one air dried tooth (premolar or molar) in each quadrant with a sterile gracey curette (Kentzler-Kaschner Dental GmbH, Ellwangen, Germany) after placing cotton roles buccally and lingually. The tip was dipped in an Eppendorf tube filled with 500 µl sterile tryptic soy broth (TSB) and the biofilm sample was transferred into the tube by gently moving the curette for five seconds. After pooled sample collection of all four quadrants, the tube was closed, and stored at 4 °C until microbiological analysis (maximal 4 h).

Professional tooth cleaning was accomplished with an air-polishing device (Airflow^®^ prophylaxis master and Airflow^®^ Plus powder; both EMS, Nyon, CH), and, if needed, with sonic scalers and rubber cups with polishing paste (Cleanic^®^, Kerr, Bioggo, CH). There was no difference in the procedure between experimental and control group.

### Oil pulling

Probands were instructed on daily oil pulling which was performed in the early morning immediately after getting up on empty stomach and before drug intake, oral hygiene, or breakfast. All subjects were asked to rinse eight weeks with their allocated liquid. The defined volume of 15 ml was dosed with measuring cups (Sensoplast Packmitteltechnik GmbH, Oberhonnefeld, Germany). The rinsing substance was orally administered, pulled, and not swallowed. After 15 min of swishing liquid all around the oral cavity from left to right, front to back and vice versa and sucking and pulling the oil through the teeth, the liquid was spit into a reservoir for waste oil or into a paper towel and put into a trashcan. Afterwards the mouth was rinsed gently with warm tap water for reducing the taste of sesame oil, followed by the probands daily oral hygiene routine [26].

### Follow-up investigations

Re-investigations were done after 28 and 56 days of oil pulling. At each appointment, the same blinded investigator (KV) assessed GBI and RMNPI as described earlier. Supragingival plaque samples were taken from the same air-dried teeth and transferred into a tube. Professional tooth cleaning was only repeated at the end of the study.

### Microbial analysis

The tubes containing the samples in 500 µl TSB were vortexed three times for 5 s and subsequently ultrasonicated for 2 min in an ultrasound water bath (Bandelin Sonorex, 35 kHz; type RK 102 H; Bandelin Electronic, Berlin, Germany) to suspend the plaque bacteria. Samples were vortexed three times for 5 s again. They were 1000-fold diluted in 0.9% sodium chloride in two steps (100 µl + 900 µl saline first, then 10 µl from this dilution + 990 µl saline). From these dilutions, 50 µl aliquots were plated on different agar plates using an automatic spiral plater (model WASP 2; Don Whitley, Shipley, West Yorkshire, UK). The detection limit, therefore, was 2 × 10^4^ colony forming units (cfu)/mL. The chosen dilution range was ideal in our study to obtain countable cfu numbers for the prevailing bacterial species in our collective of test persons. Growth media used were Columbia agar with 5% blood (Columbia III agar, number 254098, Becton & Dickinson), Schaedler agar (number 254084, Becton & Dickinson), chocolate agar (in-house Institute of Hygiene and Medical Microbiology, Innsbruck). Plates were incubated for 48 h at 37 °C in general, and the number of cfu was counted. Plates with high cfu numbers rapidly grown were counted after 24 h, plates with low cfu numbers after up to 72 h. Columbia agar and chocolate agar were incubated in 5% CO_2_ and Schaedler agar in anaerobic atmosphere.

The most frequent bacteria were identified by matrix-assisted laser desorption/ionization-time of flight mass spectrometry (MALDI-TOF MS) (apparatus Bruker Daltonics) using the direct smear method. A score above 1.7 was considered valid [27]. The bacterial species isolated and identified by MALDI-TOF MS and selected for evaluation of their counts on the different agars are shown in Fig. 3 and Suppl. Figures 1 and 2.

### Statistical methods

Sample size calculation was based on the data provided by Ripari et al. 2020 [28]. In that study, a similar study design as in the present trial was used to evaluate the effect of 4-week oil pulling with coconut oil compared to no treatment on plaque and gingival index. In that study, the mean percentage of plaque index was 19.3 ± 10.37% after 4-week oil pulling and 29.1 ± 9.06% after no intervention. Based on these data, sample size calculation for independent samples with a power of 80% and α = 0.05 revealed a sample size of seven per group. A proposed drop-out rate of 20% increased the number per group to eight. Due to minimal differences in the study design (distilled water instead of no treatment) and to increase credibility of data, the sample size was increased to 20 per group, resulting in a total of 40 probands.

Data were analyzed for normality of distribution with the Shapiro–Wilk test, not resulting in a normal distribution. Thus, the Mann-Whitney U-test was used to determine differences between the independent test and control groups in terms of RMNPI and GBI, as well as the counts of colony forming units. Comparison of pre- and post-values within the same group were done using the Wilcoxon signed-rank test and Kruskal-Wallis test with Dunn’s multiple comparisons test. P-values < 0.05 were considered as the level of significance. Data analysis was performed using SPSS software for Windows, Version 29.0.0.0 (SPSS Inc, Chicago) and, for evaluation of the microbiological results, GraphPad Prism, version 8.0.1 (Graph- Pad, Inc., La Jolla, CA, USA). The examiner who assessed the index scores and collected the plaque samples (KV), and interpreted the microbiological results (MN), and the statistician (CZ) were blinded as to the division of the groups.

To obtain an improved comparison of the course of cfu counts of the different strains over the study period between the test and control group, the Integral Method was used, which transforms the whole curve of cfu counts (log10 cfu per ml vs. time) into one value of ‘Bactericidal Activity’ (BA, log10 cfu per ml per min) [29]. The method is based on the area below the killing curve, which is calculated by addition of the areas of trapezoids between the single time points of incubation and transformed into an orthogonal triangle with the same area. Its hypotenuse forms with the abscissa the angle a, whose tangent, tga = y/2x, represents the sought average BA. The method allows an expanded statistical analysis, particularly between killing curves with small differences. The three time points of evaluation in the study were put in into the algorithm as 0, 30 and 60 min, which allowed a reliable comparison of curves. Student’s unpaired t-test was used for comparison between test and control groups.

## Results

### Study population

Forty individuals (29 females and 11 males; all Caucasians) with a mean age of 37 years (range 23–61 years) were randomly allocated either to control (13 females, 7 males) or test group (16 females, 4 males). There was a drop-out rate of 10% (three individuals in the control and one in the test group; all females) due to mucosal irritation (one person after four weeks) or vacation (two individuals at week eight).

### Rustogi modified navy plaque index

**Fig. 1:**
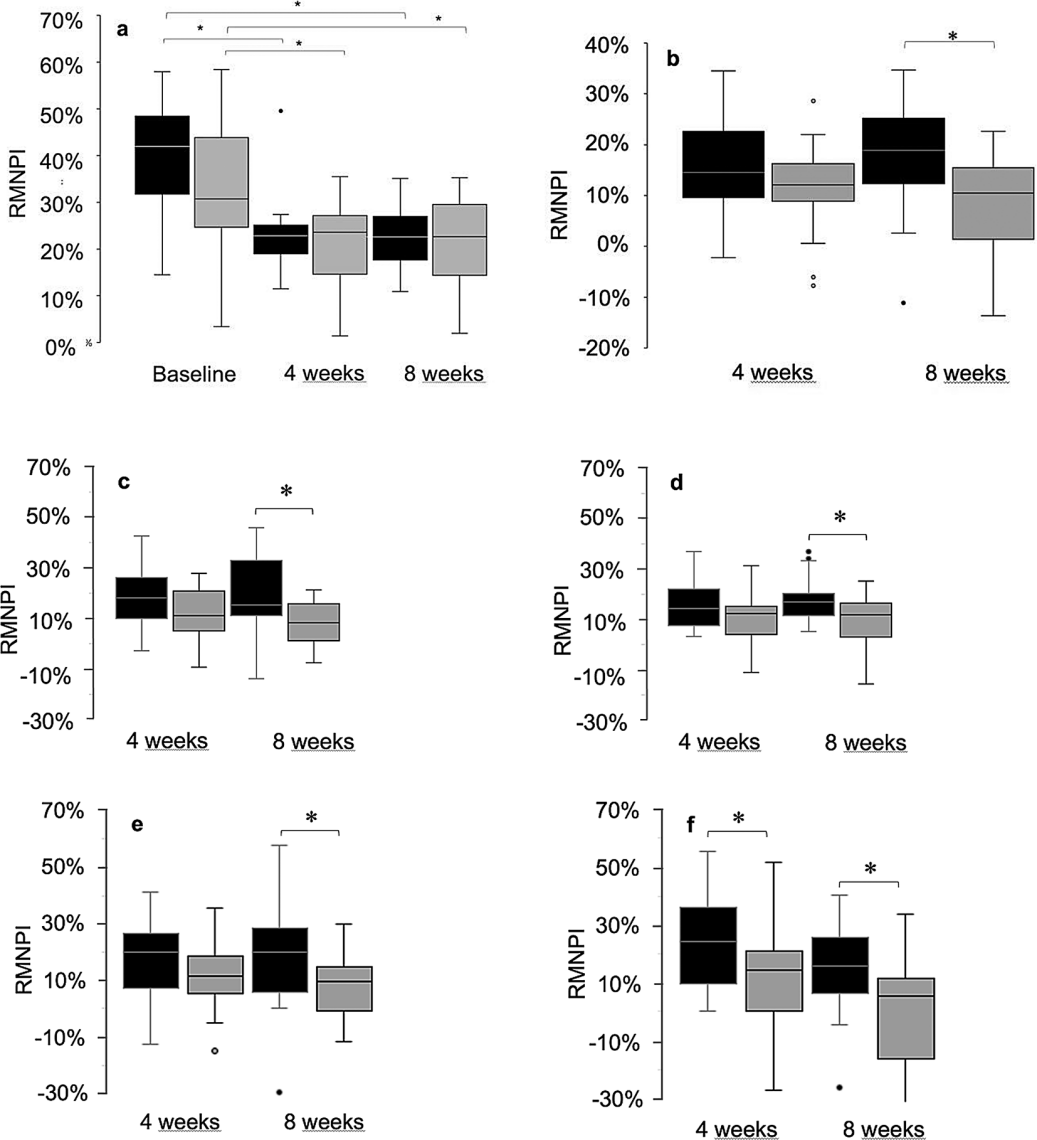
Rustogi Modified Navy Plaque Index (RMNPI, %). (**a**) Full mouth RMNPI at baseline, and after 4 and 8 weeks of pulling with sesame oil (test group – black) or distilled water (control group – grey). RMNPI between test and control was statistically significantly different at baseline (*p* = 0.046), but not after four (*p* = 0.88) and eight weeks (*p* = 0.62). The test group showed a statistically significant reduction in RMNPI from baseline to week four and week eight (*p* < 0.001). There was no further decrease in RMNPI between weeks four and eight (*p* = 0.59). The same was found for the control group with a statistically significant reduction of RMNPI from baseline to week four (*p* < 0.001) and week eight (*p* = 0.004). Again, there was no further decrease in RMNPI between weeks four and eight for pulling with distilled water (*p* = 0.74). (**b**) Reduction of full mouth RMNPI after 4 and 8 weeks of intervention. After four weeks, there was no statistically significant difference between the groups in median plaque reduction. After eight weeks, there was a significantly higher reduction of RMNPI in the test group compared to control (*p* = 0.023). (**c**-**f**) RMNPI reduction of subscales (**c**) anterior teeth, (**d**) buccal sites, (**e**) lingual, and (**f**) approximal surfaces after 4 and 8 weeks of intervention. There were no statistically significant differences between control and test group after four weeks of pulling. Statistically significant higher reduction of RMNPI in the test group was found after eight weeks of intervention in anterior (15.28 vs. 8.33%), buccal (16.89 vs. 11.90%) and lingual (20.24 vs. 9.52%) subscales. In approximal subscale, the reduction of RMNPI was significantly higher in test group after four (24.07 vs. 14.29%) and after eight (16.00 vs. 5.36%) weeks. Statistically significant differences are marked with an asterisk

At baseline there was a significantly higher full mouth RMNPI in the test group (41.42%; range 14.48–57.94%) compared to the control group (30.71%; range 3.37–57.94%) (*p* = 0.046), which was attributable to higher plaque levels in posterior, lingual and approximal subscales (for details see Table 1; Fig. 1). After eight weeks, statistically significantly higher reduction of full mouth RMNPI was found for oil pulling compared to control with 18.98% (range − 11.11–34.72%) versus 10.49% (range − 13.64–22.62%) (*p* = 0.023), and was most pronounced in anterior, buccal, and lingual subscales (Table 1). On approximal sites, the reduction of RMNPI was statistically significantly higher in the test group after four (24.07% (range 0.00–55.36%) vs. 14.29% (range − 57.14–51.79%); *p* = 0.030) and after eight weeks (16.00% (range − 26.00–40.18%) vs. 5.36% (range − 37.50–33.93%); *p* = 0.015).

**Table 1:** Rustogi modified navy plaque index (RMNPI) at baseline, and plaque reduction after four and eight weeks of pulling with sesame oil (test) or distilled water (control). Statistically significant differences were found at baseline with higher full mouth RMNPI in the test group, as well as in posterior, lingual and approximal subscales. Statistically significant higher reduction of RMNPI in the test group was found after eight weeks of intervention in full mouth and in anterior, buccal and lingual subscales. In approximal subscale, the reduction of RMNPI was significantly higher in the test group after four and after eight weeks. The highest reduction was found in marginal tooth surfaces

	Oil pulling	Control group	*p*-value
**RMNPI full mouth**			
Baseline RMNPI, %	41.42 (14.48–57.94)	30.71 (3.37–58.44)	**0.046**
Plaque reduction week 4, %	14.48 (-2.2–34.52)	12.10 (-7.74–28.60)	0.107
Plaque reduction week 8, %	18.98 (-11.11–34.72)	10.49 (-13.64–22.62)	**0.023**
**RMNPI posterior teeth**			
Baseline RMNPI, %	43.58 (17.71–60.07)	34.10 (3.82–61.11)	**0.030**
Plaque reduction week 4, %	13.54 (-1.71–31.60)	10.07 (-15.66–29.63)	0.146
Plaque reduction week 8, %	16.67 (-8.55–37.15)	12.50 (-19.70–30.21)	0.061
**RMNPI anterior teeth**			
Baseline RMNPI, %	38.66 (10.19–63.43)	30.09 (6.94–55.09)	0.18
Plaque reduction week 4, %	18.06 (-2.78–42.59)	11.11 (-9.26–27.78)	0.208
Plaque reduction week 8, %	15.28 (-13.89–45.83)	8.33 (-7.58–21.30)	**0.016**
**RMNPI buccal**			
Baseline RMNPI, %	39.84 (25.40–68.25)	29.17 (3.57–63.10)	0.081
Plaque reduction week 4, %	14.29 (3.17–36.90)	12.35 (-11.11–31.35)	0.285
Plaque reduction week 8, %	16.89 (5.15–36.90)	11.90 (-15.66–25.21)	**0.025**
**RMNPI lingual**			
Baseline RMNPI, %	40.47 (13.33–65.08)	32.34 (3.17–65.84)	**0.046**
Plaque reduction week 4, %	19.84 (-12.44–41.27)	11.51 (-14.68–35.39)	0.175
Plaque reduction week 8, %	20.24 (-29.33–57.94)	9.52 (-11.62–30.16)	**0.049**
**RMNPI approximal**			
Baseline RMNPI, %	66.37 (31.5–89.29)	49.11 (0.89–93.52)	**0.018**
Plaque reduction week 4, %	24.07 (0.00–55.36)	14.29 (-57.14–51–79)	**0.030**
Plaque reduction week 8, %	16.00 (-26.00–40.18)	5.36 (-37.50–33.93)	**0.015**
**RMNPI marginal**			
Baseline RMNPI, %	62.38 (11.41–86.67)	54.76 (9.52–83.33)	0.201
Plaque reduction week 4, %	22.62 (-19.33–49.40)	23.21 (-9.09–48.72)	0.925
Plaque reduction week 8, %	28.57 (-29.33–68.45)	23.21 (-21.97–48.08)	0.146

### Gingival bleeding index (GBI)

**Fig. 2:**
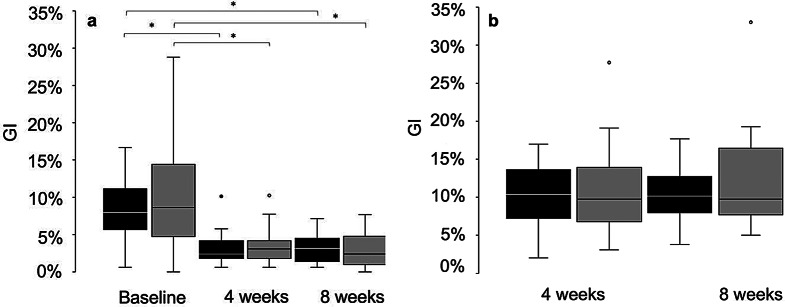
Gingival bleeding index (GBI, %). (**a**) Full mouth GBI at baseline, and after 4 and 8 weeks of pulling with sesame oil (test group - black) or distilled water (control group - grey). There was no statistically significant difference at baseline for GBI values between test and control group (*p* = 0.547). Both groups showed a statistically significant reduction of full mouth GBI after four and eight weeks of intervention compared to baseline. (**b**) Reduction of full mouth GBI after 4 and 8 weeks of intervention. In the control group there was a median reduction of total GBI of 4.76% at both times, compared to the median reduction of 5.36 (4 weeks) and 5.13% (8 weeks) in the test group. No significant differences between test and control group in the reduction of GBI were found in total GBI or subscales

There was no statistically significant difference at baseline for GBI values between test (7.99%; range 0.60–16.67%) and control group (8.63%; range 0.00–28.79%) (*p* = 0.547) (Fig. 2). For the test group, GBI was statistically significantly reduced after four (median GBI 2.38%; range 0.60–10.12%; *p* = 0.005) and eight weeks of oil pulling (3.09%; range 0.60–7.14%; *p* < 0.001). Within the control group, there was also a statistically significant reduction of GBI from baseline to week four (median GBI 3.09%; range 0.60–10.26%; *p* < 0.001) and week eight (2.38%; range 0.00–4.76%; *p* < 0.001).

There was no statistically significant difference between the test and control groups at any time in either the absolute level or the reduction in GBI, neither in full mouth GBI nor in subscale analyses (*p* > 0.05). Full mouth GBI reduction after four weeks for the test group was 4.76% (range − 1.92–22.73%) compared to control with 5.36% (-2.97–11.99%), and after eight weeks 4.76% (range 0.00–28.03%) versus 5.13% (range − 1.19–12.67%), respectively.

### Microbiological results

**Fig. 3:**
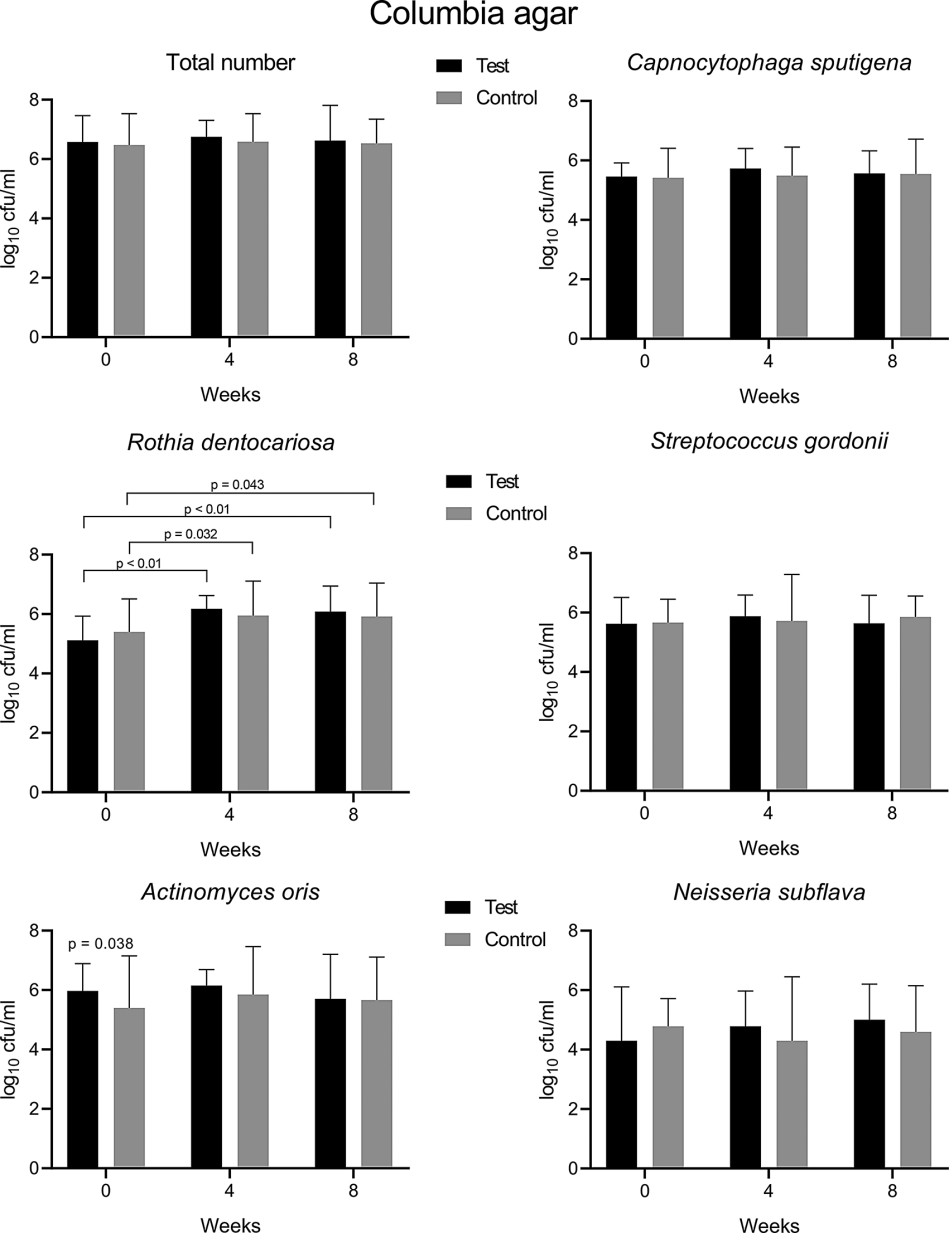
Counts of colony forming units (cfu) of bacteria grown from supragingival plaque samples on Columbia agar at baseline and after 4 and 8 weeks of pulling with sesame oil (test group) or distilled water (control). The total number includes the count of all kinds of bacteria grown on the plates. Median and range of 17 test and 20 control persons. Mann-Whitney test between pairs, p values < 0.05 are indicated. Kruskal Wallis test plus Dunn’s multiple comparison test between time points of the same group, p values < 0.05 are indicated

Eleven species from supragingival plaque samples grown in high numbers on three different agars were identified by maldi-tof-Ms. Numbers of cfu and their course over the study period are shown in Fig. 3 and Suppl. Figures 1 and 2. In general, we could not find marked differences between the test and control group. All differences between groups were minimal and remained within one log_10_ step (Fig. 3, Suppl. Figures 1 and 2). Only five scattered p values were below 0.05, for *Actinomyces oris* on Columbia agar, for the total number, *A. oris*, and *Rothia dentocariosa* on chocolate agar, and for *Veillonella parva* on Schaedler agar.

Similarly, the monitoring of the same bacterial species over time within each group revealed minimal changes at the most (Fig. 3, Suppl. Figures 1 and 2). Only the cfu count of *R. dentocariosa* in the test group increased by 1 log_10_ step in the first month from 5.12 log_10_ to 6.18 log_10_ on Columbia agar (*p* < 0.01, Fig. 1). This was less pronounced in the control group (*p* < 0.05, Fig. 1). Both findings, however, could not be confirmed on chocolate agar (Suppl. Figure 1). Significant increase by 0.6 log_10_ in the test group was found for *Leptotrichia wadei* and by 0.9 log_10_ in the control group for *A. oris* on Schaedler agar (Suppl. Figure 2).

We additionally compared the course of cfu counts of both groups using the Integral Method. The method allows to express the course and inclination of a curve over time in one value. It may disclose statistical differences of curves as a whole despite non-significant values of single time points. Only with *R. dentocariosa*, we found a small, but significantly higher increase of cfu counts in the test group compared with the control (Suppl. Table 1). All other values showed no differences between groups (Suppl. Table 1). Overall, with the Integral Method 14 out of 19 values (74%) in each group indicated a slight increase of the cfu count over time.

## Discussion

Nowadays, many patients practice oil pulling on their own initiative and are asking their dentists about its effects and benefits. As there are conflicting data in the scientific literature, the aim of this randomized, controlled, single-blind study was to strengthen the evidence.

Sesame oil was chosen in the present study for the test procedure of oil pulling as it is traditionally used in Ayurvedic medicine, and it is easy to dose. The lignans sesamin, sesamolin and sesaminol have potent antioxidative capacities [5, 6]. There was a long discussion in the author group on the control intervention. Some previous studies compared oil pulling to no intervention [28], others used chlorhexidine [17, 18, 20, 30] or non-chlorhexidine [31, 32] mouthrinses as control. We aimed to provide a proof-of-principle for any antioxidant and microbiological effects of the oil itself, so we mimicked the mechanical effect of pulling also in the control group. Distilled water has no therapeutic or chemical effect and was therefore chosen as negative control. A double-blind study design would have required another oil as control, which would have the same effect of emulsification and saponification. As each plant has its own phytotherapeutic spectrum, coconut or other edible oils may have had different outcomes, especially regarding microbial analysis.

The sample size of 20 probands in each group and the duration of eight weeks were well chosen to obtain robust results. A parallel group design was chosen for the current study. A cross-over design would have had the advantage of adjusting for home oral hygiene behavior, oil pulling conscientiousness, and predilection sites for plaque formation such as tooth position, and other confounding factors. However, a cross-over design always harbors the risk of a carry-over effect, i.e. that the first clinical phase influences the second phase. This is particularly relevant in microbiological investigations such as this one. In addition, the length of the wash out period for oil pulling is unclear.

Randomization was performed prior to the baseline examination without stratification. To compensate for the statistically significant difference in baseline plaque levels between the test and control group (median RMNPI 41.42% and 30.71%, respectively; *p* = 0.046), plaque reduction was selected as the primary outcome measure. Of course, plaque reduction may also be influenced by baseline plaque levels, as low plaque levels at the beginning of the study may not be reduced as much as higher plaque levels.

RMNPI was statistically significantly reduced after eight weeks of pulling with oil as well as with distilled water for 18.91% and 10.49%, respectively (*p* = 0.023). Especially in approximal sites, there was a statistically significant plaque reduction with either intervention (16.00 and 5.36%, respectively; *p* = 0.015). Plaque reduction may be either attributable to the mechanical effect of pulling or to a bias as study participants might have performed better toothbrushing due to the study situation. It is well known that the marginal and approximal dental surfaces are predilection sites for caries and gingivitis / periodontitis and are difficult to clean properly. In these sites oil pulling with sesame oil was markedly superior in reducing plaque levels compared to distilled water (Table 1). The literature on plaque-reducing effects of oil pulling is inconsistent, mainly because of differences in study designs, control interventions (e.g., no intervention, chlorhexidine mouthrinses, or water), plaque indices used and outcome of interest, which may be plaque regrowth or plaque reduction. Focusing on studies using (distilled) water as control, Nagilla et al. 2017 observed a statistically significantly lower plaque regrowth after seven days of coconut-oil pulling compared to control without toothbrushing [33]. Studies using chlorhexidine mouthrinses as control and sesame seed oil as test product reported no statistically significant differences in plaque indices, further strengthening the plaque reducing capacity of oil pulling [23, 34]. For example, Sezgin et al. 2019 showed a similar inhibitory activity on plaque regrowth in a randomized-controlled crossover trial of four days of oil pulling compared to chlorhexidine [17]. Further, similar results for sesame and coconut oil were described regarding plaque regrowth inhibition [35]. Matching with our results, the existing literature also describes significant reductions of gingival index scores with daily oil pulling, but no significant differences between oil pulling and control groups [23, 34]. This is especially relevant, as those studies compared daily oil pulling to chlorhexidine mouthwash.

In the present study no changes in colony forming units of aerobe and anaerobe bacteria were shown, neither in the test nor in the control group. Considering the cohort having no advanced periodontal disease, it is desirable, that oil pulling did not mess the resident flora and did not result in a dysbiotic state. Similar unremarkable microbiological effects of oil pulling on aerobic bacterial colony counts where shown by Asokan et al. 2009, who compared sesame oil with CHX in adolescents with plaque-induced gingivitis [17]. The also collected and investigated dental plaque samples. Conversely, Sood et al. 2014 detected statistically significant differences in CFUs of anaerobic bacteria in subjects with mild periodontal issues manageable with routine care between oil-pulling and placebo group and significant reductions within each group [30]. The took samples from the tongue. These facts allow the consideration, that microbiological changes by oil pulling may be found in saliva samples but not in plaque samples, where bacteria live in a stable milieu. It is noteworthy, that in these studies different parameters were investigated with different methods and thus the results are hard to compare. In accordance with the evidence, more rigorous, homogenous and better reported clinical trials are suggested [15, 23, 34].

Although oil pulling is known to have rarely side effects, except of individual cases of lipoid pneumonia [36], in our study one person had to be excluded because of mucosal abrasion. In the medical history psoriasis was noted. The excessively practiced rinsing, as reported by the subject, is known to have potential side effects [37], and could have been a mechanical overload for the existing keratosis. After stopping the intervention, the symptoms resolved within a few days. Consequently it can be concluded, that - additionally to children or subjects with mental, structural or motoric disabilities [36, 38] - individuals with different kinds of keratosis should take extra care or avoid oil pulling.

We monitored our probands regarding their subjective perception of oil-pulling. Compared to the control group, after eight weeks of intervention the subjects of the test group reported more often about improvements of symptoms such as mouth dryness, little saliva, sticky saliva, bad taste in mouth and subjective sensation of bad breath, for which the differences were statistically significant. Further, we supervised our participants to identify potential problems with oil pulling. Neither in the test nor control group cases of aspiration or accidental ingestion were reported. There was no significant difference in the urge to gag between the groups after four weeks, which equally decreased at the end of study duration. Most individuals reported about a better mouthfeel and almost all participants would like to continue with practicing oil pulling as a permanent ritual in their daily life.

The number of systematic reviews concerning oil pulling published in the last years shows the relevance and the currency of the topic. Patients love additional measures for oral hygiene at home, which have a health-promoting effect. However, in our opinion, in vitro studies on the principle of oil pulling are lacking. Bias in in vivo studies can never be completely ruled out due to different baseline situations and possible influences of the study situation per se, even if the test subjects were very strictly selected and monitored, as in the present study. Mechanical and therapeutic effects of different oils should be investigated on in vitro biofilms to provide the scientific evidence. Further clinical trials could focus on immunological markers like aMMP-8, sIL-6R, calprotectin, IL-6, TNF-α, IL-1 in people without advanced periodontal disease and periodontally severe diseased people to finally clarify the evidence.

## Conclusion

In summary, it can be stated that plaque reduction was statistically significantly higher with oil pulling than with distilled water, however, a study bias cannot be ruled out. Oil pulling had no additional effect on gingival health and the microbiological composition of the biofilm. Thus, oil pulling may be recommended as an adjuvant to mechanical dental cleaning for plaque reduction.

## Electronic supplementary material

Below is the link to the electronic supplementary material.

Supplementary Material 1

Supplementary Material 2

## Data Availability

No datasets were generated or analysed during the current study.
