# Supplementary material for: The plaque reducing efficacy of oil pulling with sesame oil: a randomized-controlled clinical study
Source: Clin Oral Investig. 2025 Jan 9;29(1):53. doi: 10.1007/s00784-024-06134-y (PMC11717832; doi:10.1007/s00784-024-06134-y)
Supplement: Supplementary file 1 — Supplementary Material 1 [file 784_2024_6134_MOESM1_ESM.pdf]

Suppl Table 1 BA (‚Bactericidal Activity’) values of oil pulling over 8 weeks

	Columbia agar		Chocolate agar		Schaedler agar	
Species	Test	Control	Test	Control	Test	Control
Total number	-0.0130 ± 0.0063	-0.0014 ± 0.0076	0.0022 ± 0.0067	0.0035 ± 0.0079	-0.0005 ± 0.0064	-0.0022 ± 0.0077
Actinomyces oris	0.0005 ± 0.0077	-0.0034 ± 0.0093	-0.0007 ± 0.0078	0.0000 ± 0.0098	-0.0045 ± 0.0152	-0.0101 ± 0.0150
Rothia dentocariosa	**-0.0135 ± 0.0078** [a]	**-0.0069 ± 0.0084**	**-0.0034 ± 0.0099** [b]	**0.0033 ± 0.0126**		
Neisseria subflava	-0.0037 ± 0.0123	-0.0017 ± 0.0167	-0.0043 ± 0.0112	-0.0006 ± 0.0138		
Streptococcus gordonii	0.0005 ± 0.0080	-0.0014 ± 0.0095			-0.0068 ± 0.0144	-0.0053 ± 0.0152
Capnocytophaga sputigena	-0.0016 ± 0.0083	-0.0021 ± 0.0100				
Streptococcus sanguinis			0.0014 ± 0.0079	0.0000 ± 0.0093		
Streptococcus anginosus			-0.0002 ± 0.0146	0.0017 ± 0.0118		
Haemophilus parainfluenzae			-0.0002 ± 0.0141	-0.0012 ± 0.0165		
Leptotrichia wadei					-0.0068 ± 0.0169	-0.0002 ± 0.0152
Veillonella parvula					0.0004 ± 0.0091	-0.0018 ± 0.0122
Streptococcus oralis					-0.0058 ± 0.0113	-0.0074 ± 0.0122

BA - 'Bactericidal activity' as a measure for the inclination of the curve of colony counts over time according to the Integral Method.
Mean values ± SD of 17-20 test persons; negative values indicate an increase in cfu counts, positive values a decrease.
$p > 0.1$ between test and control groups except for the following values by Student's unpaired *t* test.

[a] $p = 0.014$ versus control

[b] $p = 0.069$ versus control
