# Supplementary material for: The plaque reducing efficacy of oil pulling with sesame oil: a randomized-controlled clinical study
Source: Clin Oral Investig. 2025 Jan 9;29(1):53. doi: 10.1007/s00784-024-06134-y (PMC11717832; doi:10.1007/s00784-024-06134-y)
Supplement: Supplementary file 2 — Supplementary Material 2 [file 784_2024_6134_MOESM2_ESM.pdf]

Chocolate agar

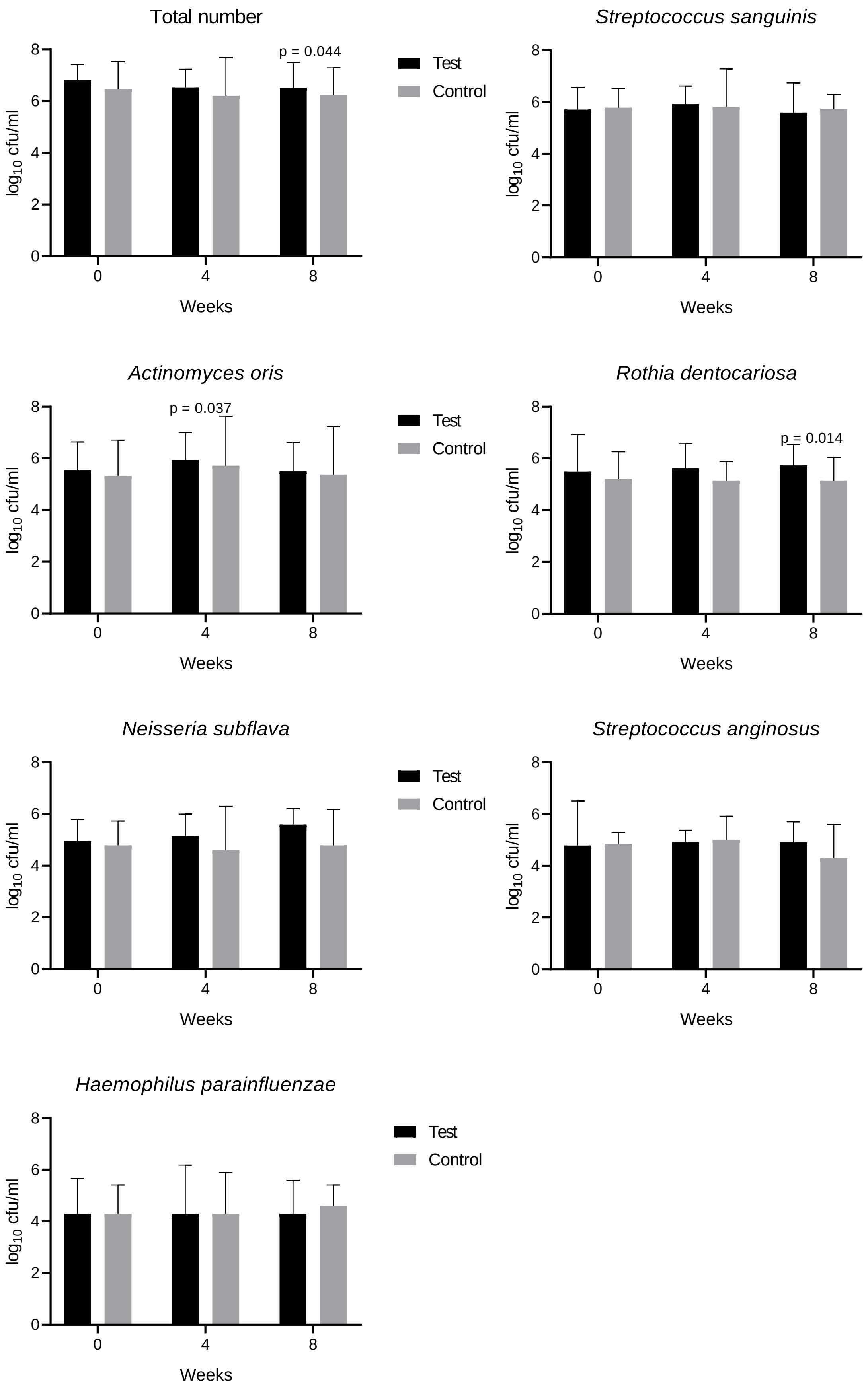

Suppl. Fig. 1 Counts of colony forming units (cfu) of bacteria grown from supragingival plaque samples on *chocolate agar* at baseline and after 4 and 8 weeks of pulling with sesame oil (test group) or distilled water (control). The total number includes the count of all kinds of bacteria grown on the plates. Median and range of 17 test and 20 control persons. Mann-Whitney test between pairs, p values < 0.05 are indicated. Kruskal Wallis test plus Dunn's multiple comparison test between time points of the same group, p values < 0.05 are indicated.

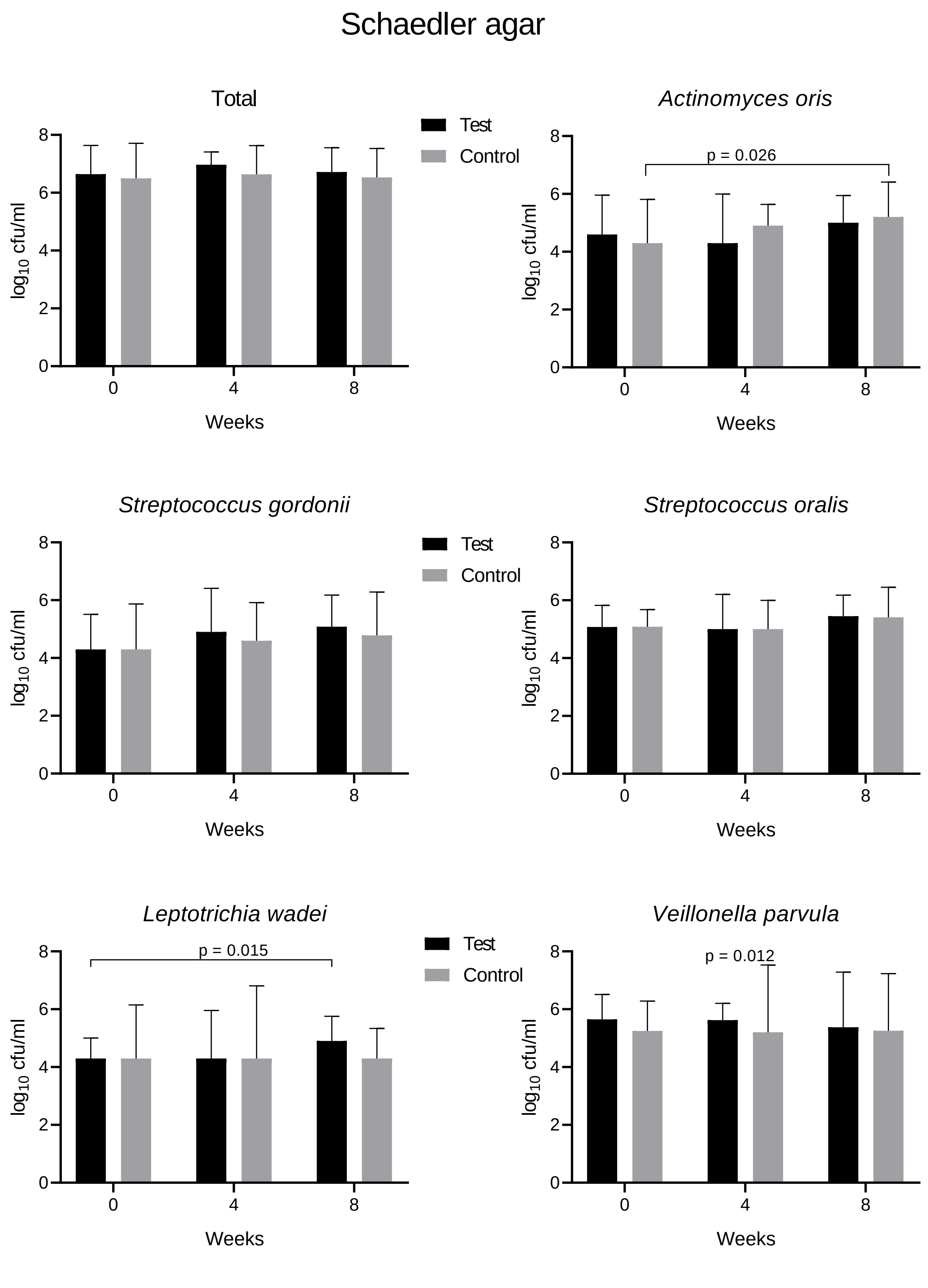

Suppl. Fig. 2 Counts of colony forming units (cfu) of bacteria grown from supragingival plaque samples on Schaedler agar at baseline and after 4 and 8 weeks of pulling with sesame oil (test group) or distilled water (control). The total number includes the count of all kinds of bacteria grown on the plates. Median and range of 17 test and 20 control persons. Mann-Whitney test between pairs, p values < 0.05 are indicated. Kruskal Wallis test plus Dunn's multiple comparison test between time points of the same group, p values < 0.05 are indicated.
